# Post–*Modern Epidemiology*: When Methods Meet Matter

**DOI:** 10.1093/aje/kwz064

**Published:** 2019-03-16

**Authors:** George Davey Smith

**Affiliations:** Medical Research Council Integrative Epidemiology Unit, Bristol Medical School, University of Bristol, Bristol, United Kingdom

**Keywords:** Bradford Hill, causal inference, history of epidemiology, liability models, methodology, stochasticity

## Abstract

In the last third of the 20th century, etiological epidemiology within academia in high-income countries shifted its primary concern from attempting to tackle the apparent epidemic of noncommunicable diseases to an increasing focus on developing statistical and causal inference methodologies. This move was mutually constitutive with the failure of applied epidemiology to make major progress, with many of the advances in understanding the causes of noncommunicable diseases coming from outside the discipline, while ironically revealing the infectious origins of several major conditions. Conversely, there were many examples of epidemiologic studies promoting ineffective interventions and little evident attempt to account for such failure. Major advances in concrete understanding of disease etiology have been driven by a willingness to learn about and incorporate into epidemiology developments in biology and cognate data science disciplines. If fundamental epidemiologic principles regarding the rooting of disease risk within populations are retained, recent methodological developments combined with increased biological understanding and data sciences capability should herald a fruitful post–*Modern Epidemiology* world.

In Jerry Morris’ seminal (but now largely unread) 1957 book *The Uses of Epidemiology*” (1), he identified 7 uses of epidemiology, one of which was discovering the causes of disease (2). In this brief commentary, I will focus entirely on etiological epidemiology and provide an impressionistic account of how the tension between methodological development and real-world investigation has played itself out in the field of noncommunicable disease epidemiology.

## THE SEEDBED OF MODERN EPIDEMIOLOGY

Epidemiology in the mid-20th century was coming to terms with the transition from communicable to apparently noncommunicable diseases in high-income countries. Morris modestly suggested his book should have been entitled “Some uses of epidemiology in the study of non-communicable disease” (1, p. V). The 1960 text *Epidemiologic Methods* by MacMahon, Pugh, and Ipsen (3) similarly reflected a shift from communicable disease epidemiology—the primary focus of earlier pioneering epidemiologic textbooks by Major Greenwood (4) and Taylor and Knowelden (5)—to investigation of noncommunicable disease.

A third of the first edition of Morris’s book was devoted to the search for causes of disease, and he highlighted multiple causality as likely being at the root of the chronic, apparently noncommunicable, diseases under investigation(1). Joint analysis of the increasing number of putative risk factors for coronary heart disease (CHD) was widely adopted following Cornfield’s use of Fisher’s discriminant function in the multivariable—then generally referred to as multivariate (6)—setting in 1962 (7). Morris applied this to the investigation of physical activity in the seminal London busmen study in 1966 (8), with Cornfield et al. (9) presenting a detailed analysis of Framingham data in 1967. This was largely superseded by the closely related multiple logistic regression (10), which rapidly became ubiquitous in the epidemiologic sphere.

Almost as quickly as they were taken up, such effortless off-the-shelf approaches to identifying “independent” risk factors were decried. Murphy considered that “[m]ultivariate analysis (which in certain quarters is being substituted for scientific perception), can spread its soporific effect” (11, p. 1860) and that (with respect to some analyses) “I am driven to believe that however excellent the prediction, the formula, from an etiological and ontological standpoint, provides no insights whatsoever” (11, p. 1860). Leaders of the field joined in. Reuel Stallones opined that then-contemporary epidemiology demonstrated a “continuing concern for methods, and especially the dissection of risk assessment, that would do credit to a Talmudic scholar and that threatens at times to bury all that is good and beautiful in epidemiology under an avalanche of mathematical trivia and neologisms” (12, p. 69). Abe Lilienfeld thought that “[p]erhaps the most dangerous aspect of the state of our discipline today is that there is an unhealthy emphasis on how one conducts an epidemiologic study and not why and what one does in such a study. Simply put, we are training *technocrats*” (13, p. 147).

## EPIDEMIOLOGY ENCOUNTERS MODERNITY

From the early 1970s onward, a series of papers interrogating the fundamental tenets of epidemiology appeared. Among the many authors, Olli Miettinen (14–20) and Ken Rothman (21–25) were particularly influential early contributors. Rothman’s 1986 book *Modern Epidemiology* (26) represented a watershed moment in the discipline. Improving the ability to identify causes of disease was naturally a major concern of this rebooting of epidemiologic methodology. For example, in a 1974 paper on “Synergy and antagonism in cause-effect relationships” (21), Rothman advanced the notion that synergy was represented by supra-additive effects (referred to as “biologic interaction” (27, 28)), an approach that would “provide clues to the behavior of the causal mechanisms involved” (21, p. 386).

Morris’s *Uses of Epidemiology* (1) opened with the presentation of population data on disease trends (e.g., CHD, peptic ulcers, and lung cancer) that urgently required improved etiological understanding so that prevention activities could be mounted. Throughout the book, he discussed many other situations (from cancers through occupational illnesses to the changing socioeconomic and sex ratios in morbidity and mortality rates), with epidemiologic methodology being discussed implicitly in terms of how it could be applied to these concrete issues. The meat of *Modern Epidemiology* (26), in contrast, commenced with a largely abstract chapter entitled “Causal Inference in Epidemiology” that advanced a Popperian philosophy, expanded on Rothman’s deterministic “causal pies” model (22), and critiqued what it referred to as Bradford Hill’s (29) criteria for causal inference (26). *Modern Epidemiology* represented an epistemic break in the discipline and established a set of generally accepted axioms that few have questioned. My pirated photocopy (the book was expensive) is, from start to finish, heavily annotated and proves at this distance how much I encountered for the first time and learned from reading it.

In the year that *Modern Epidemiology* was published, the start of increasingly formal and mathematized causal inference in epidemiology was heralded by Jamie Robins’s brilliant work introducing graphical causal modelling and the g-formula/G-estimation framework (30) and influential papers by Robins and Sander Greenland (31, 32) in which they connected causal inference to epidemiologic analysis. These demonstrated their utility in subsequent studies within the field of human immunodeficiency virus/acquired immunodeficiency syndrome.

The rumbling dissatisfaction of some senior epidemiologists continued, pointing out an apparent increasing disconnect between a methodology-obsessed epidemiology and the fruitful investigation of patterns of the causes of disease within populations. Three contributions from 1988 are illustrative. Leon Gordis sensed an increasing disconnect between epidemiology and biology and referred to epidemiologic studies being “considered ‘positive’ only because they use highly sophisticated statistical techniques that have become available only in recent years” (33, p. 2). Diana Petitti reported that within epidemiology, she had found “less and less evidence of scientific creativity and more and more striking deficits in the understanding of biology” (34, p. 149), with the epidemiologic literature becoming “an archive of the results of information derived from mechanical applications of multivariate analysis” (34, p. 150). Jerry Morris (who was, as we have seen, an early adopter of methods when he saw them as useful) reported that he had “high regard for Rothman’s *Modern Epidemiology*” (35, p. 100) but that “as a guide to modern epidemiology the book has serious limitations” (35, p. 100).

“The student coming to it afresh could not gather that epidemiology is the basic science of public health. Thus in close on 150 years of epidemiological research (Dr Rothman doesn’t have much space for history) it continues plausible that the main determinants of the health of populations and sizable subgroups in them are their economic-social-cultural conditions. The data on this are mostly cross-sectional and inevitably derived from studies of populations and groups as the unit, rather than from aggregation of individuals with their various attributes” (35, p. 100).

## THE MODERN VIEW OF CAUSALITY

Morris (1, 35, 36) (and others, such as Mervyn Susser (37, 38)) saw causality as inherent in underlying sociocultural processes working through mediating factors to influence the health of individuals within populations; ultimately, epidemiologic causality needed to be considered as a population phenomenon that could often be usefully interrogated through individual-level investigation. *Modern Epidemiology* (26) and its fellow travelers had a different view. I (probably unfairly) previously characterized this as one in which “. . . the health of populations has become a footnote to a detailed exposition of how to calculate a multivariably adjusted effect estimate from a study with appropriate sampling, and then how to apply a billiard-ball view of causation to your study results” (2, p. 1148).

*Modern Epidemiology* (26) incorporated Rothman’s notion of “biologic interaction,” one that surely would have failed to satisfy calls from Gordis and others to engage seriously with biology. Despite its name, biologic interaction makes little concession to actual biology (39) and is far removed from the obvious deep engagement with the basic sciences seen in earlier work on actual models of disease development, such as in the work of Richard Peto (40). Indeed, if this commentary has the effect of engaging more contemporary epidemiologists in reading such contributions—the referenced paper introduced the fascinating “Peto’s paradox” (41)—it has been worth writing.

Biologic interaction was advanced as a route to causal identification, but it is hard to come up with many examples of the application of this model having resulted in such. The deterministic “causal pies” were contrasted in *Modern Epidemiology* (26) with potential stochastic models, and here I think it is worth reflecting on the analogous situation with different formulations of liability models developed in classic quantitative genetics (and which E. A. Murphy, who we earlier heard complain about soporific multivariate analyses, attempted to introduce to epidemiologists (42)). Falconer’s influential threshold model (43) envisages an underlying normally distributed liability in which those above a certain level are doomed to develop disease (though the confusion of the implicit concepts of incidence and prevalence in the initial paper would irritate epidemiologists). This is explicitly a deterministic model. However, the liability includes all the known and unknown nongenetic factors, the latter of which includes the nonshared environment (NSE) (44). NSE is estimated by subtraction in quantitative genetic studies (e.g., twin studies), as the residual variance after the estimated genetic component of variance and the component of variance due to shared environment—the environmental influences that lead to siblings (and others) brought up in the same home environment being similar to each other—have been subtracted from 100%. For the large majority of human traits, including diseases such as cancers, the so-called NSE is the major component of variance (45, 46). In many nonhuman forms of life, precisely the same situation is seen when decomposing the variance in a phenotype contributed to by genes and environment (45). When studying transmission of skin patterning in guinea pigs, Sewall Wright said these effects “must be due to irregularities in development due to the intangible sort of causes to which the word chance is applied” (47, p. 545). So-called NSE in human phenotypes will be driven by everything from stochastic nonperfect quantitative cytoplasmic sharing during cell division, somatic mutations, random mitotically stable epigenetic changes through to idiosyncratic life events of all types, measurement error, and (probably importantly) reverse causal influences on phenotype of developing disease (45, 48, 49). Thus, a deterministic model could be proposed because it explicitly contained the intangible variance that quantitative genetics identified as largely stochastic. An alternative model, advanced by Edwards (50), had no threshold, instead proposing an increasing probability of disease with increasing genetic liability. Probability at a given liability would thus depend upon a mixture of known and unknown (including potentially stochastic) nongenetic factors.

As will be appreciated, the Falconer and Edwards models are largely equivalent (as Rothman hints at with respect to the deterministic and stochastic causal models he considers (28)). The explicit reason Rothman gives for favoring deterministic models is that:

“In our ignorance of these hidden causal components, the best we can do in assessing risk is to assign the average value to everyone exposed to a given pattern of known causal risk indicators. As knowledge expands the risk estimates assigned to people will approach one of the extreme values, zero or unity”. (26, p. 12)

**Figure 1.**
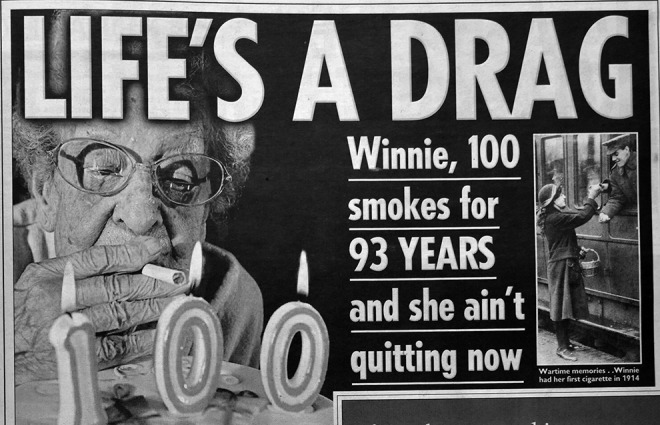
“The chance events that contribute to disease aetiology can be analysed at many levels, from the social to the molecular. Consider Winnie (Figure 1); why has she managed to smoke for 93 years without developing lung cancer? Perhaps her genotype is particularly resilient in this regard? Or perhaps many years ago the postman called at one particular minute rather than another, and when she opened the door a blast of wind caused Winnie to cough, and through this dislodge a metaplastic cell from her alveoli? Individual biographies would involve a multitude of such events, and even the most enthusiastic lifecourse epidemiologist could not hope to capture them [54]. Perhaps chance is an under-appreciated contributor to the epidemiology of disease” (45, p. 547). This photo of Winnie Langley, who smoked for 93 years, appeared in The Sun (138) and was reprinted in the *International Journal of Epidemiology* (45) Reprinted with permission.

**Figure 2.**
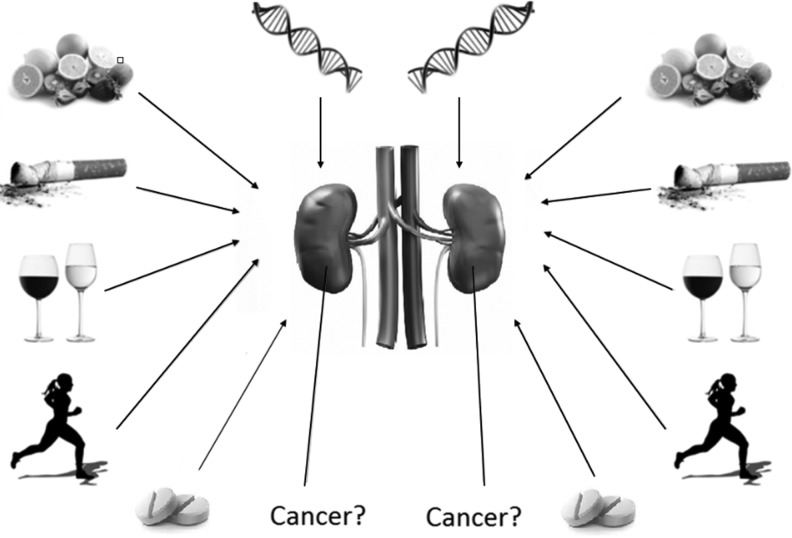
The major contribution of stochastic events and the bounds to personalized medicine is illustrated by cancers of bilateral organs.

Such a formulation—that with increasing knowledge we can approach certainty—is, of course, the epitome of the overhyped “personalized medicine,” for which much epidemiologic and other evidence suggests there are serious (and sometimes insurmountable) constraints (45, 51). In reality, it matters not whether we consider our ignorance to be ontological (are there truly stochastic processes leading to disease that are inherently unpredictable?) or epistemological (that it is simply infeasible that adequate data could be collected to identify the individual-level causal processes); basic epidemiologic and biological reasoning presents serious bounds to approaching (or even nearing) the holy grail of zero or unity values for risk (45) (Figure 1). Consider the development of cancers in bilateral organs, for example the breast (52) or kidney (53) (Figure 2). At the time when a primary cancer develops in one organ, the contralateral one will have an identical germline genotype and will have experienced exposure patterns essentially the same as those experienced by the affected organ. Despite this, the risk of developing a second primary tumor in the contralateral organ is little elevated over population risk (52, 53). For example, a monozygotic twin with a primary breast cancer has roughly half of the risk of developing a second primary cancer as her twin has of developing a first primary cancer (her twin has 2 unaffected breasts at risk as opposed to 1 in the initially affected twin). A fantasy lifecourse study in which regular tissue biopsies are obtained from early embryonic stages onwards and there is minute-by-minute monitoring of every action and exposure wouldn’t be quite intensive enough, it seems (54). However, although we can engage in fantasies of deterministic causal attribution, we should recognize that by ignoring the constraints imposed by how the material world is, we encourage the mythopoetics of personalized medicine. Epidemiologists, surely, should be suspicious of such.

Away from the abstractions on causal and deterministic models, the discussion of Hill’s (29) informal approach to strengthening causal inference in *Modern Epidemiology* (26) was not positive. It suggested some of the so-called “criteria” (26, p. 17) (Hill neither used the term nor endorsed its obvious implications) were either straightforwardly both “wrong” (26, p. 19) and “useless and misleading” (26, p. 18) (with respect to specificity) or, at best, “saddled with reservations” (26, p. 19). Popper was clearly the man.

## POST–*MODERN EPIDEMIOLOGY*: WHAT GOES ON?

The developments in epidemiologic methodology reflected in and influenced by the publication of *Modern Epidemiology* (26) might have been expected to increase the standing of the discipline as a scientific enterprise. Indeed, Rothman ended the first chapter of his book on the optimistic note that epidemiology was becoming increasingly respected and seen as part of biological science:

“Epidemiology has established a toehold as a scientific discipline. Whereas epidemiologic results were once greeted mainly with skepticism, they are now generally accorded some degree of respect. At midcentury, epidemiologists had trouble persuading the scientific community of a relation between smoking and lung cancer. By 1984, the situation had changed so much that a weak epidemiologic association observed between beta-carotene and cancer occurrence was the stimulus for a biochemical hypothesis on anti-oxidants, which was published in *Science*. The paper begins with the observation that ‘[E]pidemiological studies indicate that the incidence of cancer may be slightly lower among individuals with an above-average intake of beta-carotene and other carotenoids [55].’ The respectability evinced by this integration of epidemiology into the fold of the biologic sciences stems in large part from the emergence of a clearer understanding of the epidemiologic concepts that have become the basis of modern epidemiology” (26, p. 5).

Modern epidemiologic concepts were all set to herald in a glorious age of ever-increasing respectability and reliability. Sadly, the reverse proved to be the case: Over the years following the publication of *Modern Epidemiology*, an unprecedented outpouring of disdain for epidemiology appeared (56–61). The cause of this is foreshadowed in Rothman’s optimism: He used the example of epidemiologic evidence that β-carotene would reduce cancer risk as an example of its increasing respectability. What followed was a deluge of randomized controlled trials across a range of (in particular dietary) exposures that failed to to show that epidemiologic evidence usefully identified protective factors—including, among others, vitamin E and C supplementation and cardiovascular disease, selenium supplementation and prostate cancer, and Rothman's pin-up of β-carotene and cancer (62). In 1998, the second edition of *Modern Epidemiology* appeared (63), now with 2 principal authors and several chapters on applied epidemiology. The initial chapter gained an author and a word in its title (it was now “The emergence of modern epidemiology”) but was otherwise virtually unchanged from the first edition, except for the simple deletion of the final paragraph reproduced above (64).

**Figure 3.**
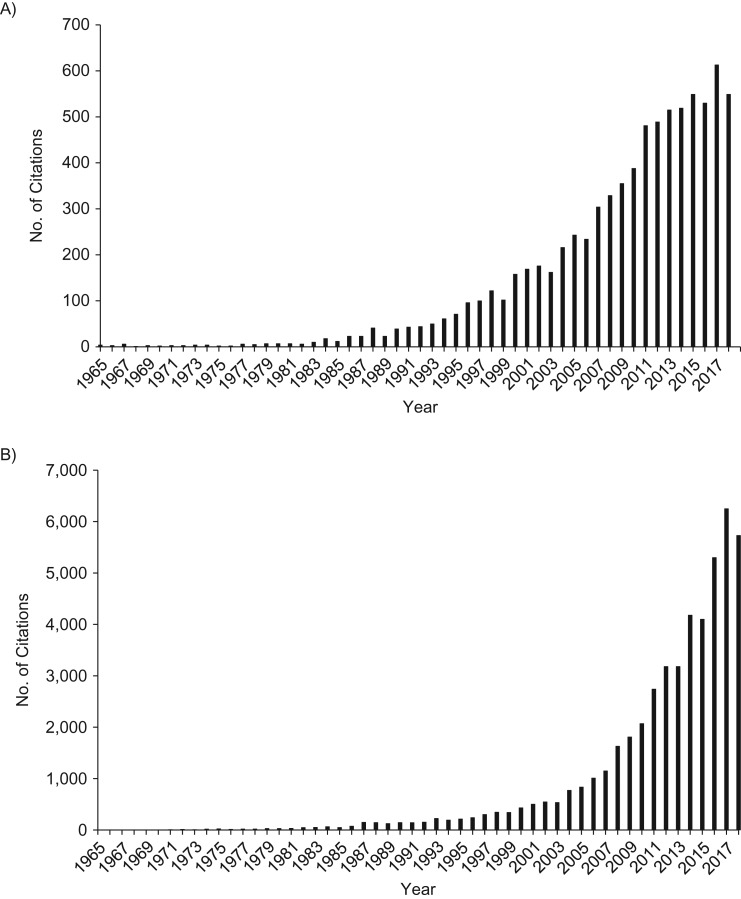
Number of Google Scholar citations from 1965 onward of Austin Bradford Hill’s seminal proto-triangulation paper “The Environment and Disease: Association or Causation?” (29) (A) and “causal inference” and “epidemiology” (B). Data from 2018 are preliminary and probably incomplete.

**Figure 4.**
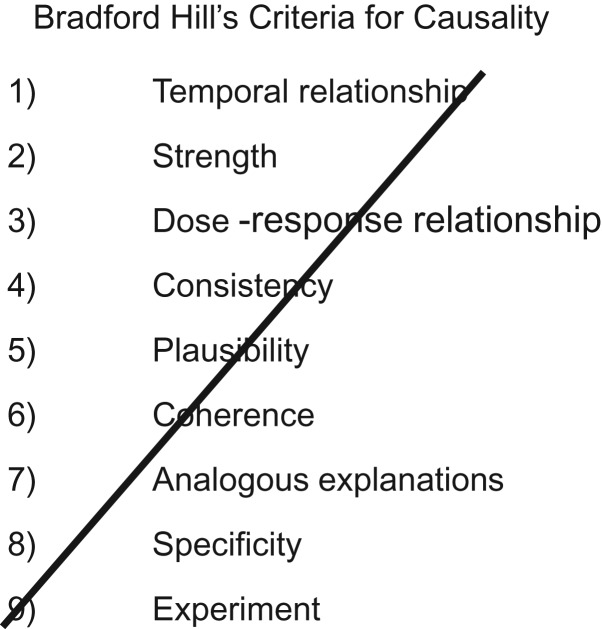
An indicative powerpoint from a recent talk on causal inference (78).

The post–*Modern Epidemiology* period has been characterized by embracing the formal language and graphical representations of the causal inference movement (65, 66). This has been unequivocally positive in many ways, in particular with respect to making transportable across particular situations the general structure of biases, for example of those due to conditioning on what is now referred to as a collider (67–70). The discipline of formally presenting proposed causal hypotheses (for exposures of interest, confounders, and nonconfounding potential covariates) is similarly helpful. However, this is only within a framework of assessment of the evidence across as many domains as can usefully provide independent evidence, whether through quantitative orthogonal evidence factors that could be combined (71) or as a broader exercise in triangulation of evidence (72, 73). Hill’s viewpoints as well as the similar set of arguments seen in the 1964 Surgeon General’s” report (74), provided prototypes for such triangulation (29, 75) but bizarrely became the target of the modernists. Labarthe and Stallones (76), in an entertaining contribution to a 1988 symposium on causal inference, ironically referred to Hill as “the villain” and correctly inferred that his contribution to actual, real-world epidemiologic inference would be greater than that of the then-hero, Popper. Citations of Hill’s work and causal inference have appropriately risen together (Figure 3). The denigration of Hill and his co-thinkers in the causal inference school remains a constant, however. In the foundational 1993 text *Causation, Prediction and Search* (77), Spirtes et al. opined that “the “epidemiological criteria for causality” were an intellectual disgrace and the level of argument . . . was sometimes more worthy of literary critics than scientists” (76, p. 302). Figure 4 was presented in an introduction to causal inference in 2018 (78), with the explicit message being that once you could draw a directed acyclic graph you no longer needed Hill and his sad, time-expired empiricism. The irony that Hill's specificity, which Rothman characterized as “wrong, useless and misleading” (26, pp. 18–19), is the basis of the now-lauded “negative controls” (79, 80) will not be lost on those familiar with earlier uses—from interrogating midcentury occupational exposures (discussed by Greenwood in 1948 (81)) through the many subsequent applications (71). Unsurprisingly, once legitimized, negative controls (aka specificity) have been overformularized and used to “correct” effect estimates (82), rather than play a more modest (but useful) role in causal inference (83).

Causal inference centered on a partially quantified “triangulation” (72, 73, 84)—based mainly on the power of study design, not analysis—should be explicitly (and prospectively) aimed at gathering evidence from approaches in which biases will be as near-orthogonal as is possible. This includes the much-despised “ecological” or population data, a combination of which allowed Fritz Lickint in 1935 to confidently declare smoking a cause of lung cancer (85). This was a quarter of a century before Cornfield’s justly celebrated and massively more systematic triangulation (with sensitivity analysis) (86), which is now increasingly seen as foundational within epidemiology.

## WERE WE EVER “MODERN”?

I started working as a chronic disease epidemiologist in the mid-1980s, around the time *Modern Epidemiology* (26) appeared, and the key questions at that time included:

Was high-density lipoprotein (HDL) cholesterol protective against coronary disease?
Why was the incidence of stomach cancer declining?
What was the major etiological factor in cervical cancer?
Could alcohol consumption protect against CHD?
Was inflammation important in cardiovascular disease?
Did antioxidants reduce the risk of cancer and cardiovascular disease?
What caused peptic ulcers?
Did higher triglyceride levels increase CHD risk?

Looking at these today, we have a much better idea about all of them; however, the contribution of observational epidemiology, ancient or modern, has been modest at best. Several turned out to have an infectious basis. In 1989, Melissa Austin predicted with respect to triglycerides, HDL cholesterol, and CHD that the answer “must come from the biological sciences” (87, pp. 256–257). In 1991, the impossibility of epidemiologic investigations making meaningful statements about causality in the HDL cholesterol/triglycerides field was advanced on statistical grounds (88), although consensus was then hardening that the epidemiologic evidence indicated that HDL cholesterol was protective (“good cholesterol”) and triglycerides an innocent bystander (89). Only randomized controlled trials (RCTs) to raise HDL (at the cost of hundreds of millions of dollars) and Mendelian randomization (90) studies (which were rather less expensive) indicated that circulating HDL cholesterol levels were, in themselves, noncausal (89). Similar stories could be told regarding others in the above list of the key questions from the mid-1980s.

The “epidemic” (60) of epidemiologic reports of “risk factors” for disease from studies that cannot realistically contribute to causal understanding has continued unabated, although now many of these are apparently examples of thoroughly modern epidemiology, being accompanied by a directed acyclic graph and the approved causal inference language (see box 3 in Krieger and Davey Smith (91) for an analysis).

The lack of any sense of accountability within epidemiology is striking. Consider 2 back-to-back papers from the *New England Journal of Medicine* in 1993 in which substantially lower risk of CHD was demonstrated among individuals using vitamin E supplements (92, 93). These were a media triumph (e.g., the *New York Times* headline “Vitamin E greatly reduces risk of heart disease, studies suggest” (94) clearly reflected causal claims). Unlike most published epidemiologic research, these studies were consequential; the use of supplements containing vitamin E among US adults increased substantially from the period before these high-profile papers appeared to the period after, with more than one third of adults taking such supplements around the turn of the century (95). Sadly the public had been misinformed, which became clear as RCT after RCT of vitamin E supplementation reported no cardiovascular benefit (96); however, it took many years for this to influence usage (97). It should be noted that in this case, the exposure being investigated in the observational studies—taking vitamin E supplements—was precisely the exposure investigated in the RCTs (randomization to taking vitamin E supplements) and that in the observational studies, apparent benefit was seen within a few years of use. Thus, the usual special pleading that the observational studies and the RCTs were not testing the same hypothesis cannot be advanced on this occasion. The investigators have never attempted to report analyses aimed at understanding why their methods produced such misleading findings. In another setting in which their findings apparently conflicted with RCT evidence (hormone replacement therapy), the original investigators have collaborated with others on a methodology that suggests there may be no disagreement between observational and RCT evidence (98). Why has this not been applied to the vitamin E case?

The scenario above (which resonates with the last paragraph from the first chapter of *Modern Epidemiology* being simply dropped) illustrates the need for epidemiology to become an open discipline, continuously reflexive and aiming to learn from experience. The two 1993 vitamin E papers, which I found unbelievable, stimulated me to write (in 1994) an editorial on “Increasing the accessibility of data” (99), as it seemed the only way accountability could be ensured was to make data available to other investigators (99). Remarkably, studies receiving mainly public funding can, a quarter of a century on, still survive without making their data available in a useful way. In the UK a series of studies—the Avon Longitudinal Study of Parents and Children (ALSPAC) (100), UK Biobank (101), and Born in Bradford (102), among others—have surely been exemplary in promoting data accessibility. Conflicts of interest are substantial within epidemiology, as Neil Pearce has laid out (103), and, as Greenland discusses (104), these are not just corporate. Cognitive and financial conflicts of interest can co-exist, for example, when research depends upon a heavily promoted methodology and the researchers do not want to revisit the occasions when these methods publicly fail nor allow others to do so. Hopefully, epidemiologists will collectively make it clear that such practices are not welcome within our discipline.

It was sobering, to me at least, to face the fact that some important epidemiologic questions that the field struggled with when I entered it were simply unanswerable by conventional epidemiologic methods. The advances that would allow some of these questions to receive better answers today using epidemiologic approaches are, in particular, due to the ability to incorporate the stunning developments in biology into an epidemiologic framework. As epidemiologists, our task is to ensure that this undoubtedly game-changing progress remains embedded within a population-sciences framework. Allowing the apparent autonomy of biological processes to go unchallenged underlies regrettable trends, from the new “polygenic eugenics” through to overpersonalized medicine. A return to pre-*Modern* epidemiologic theory, with its focus on population aggregates in actual rather than hypothetical peoples, can help keep us grounded (1, 2, 105).

This commentary relates to investigation of the etiology of disease, but in pre-*Modern* epidemiology, it was recognized that cognizance of distributions of disease was a necessary part of causal inference. Thus, Morris noted that the then-dominant theory that peptic ulcer was caused by stress was simply incompatible with the population trends and distribution of the disease (36). He was, of course, proved right (2), and identifying the primary causal agent as a treatable bacterial infection has probably considerably reduced collective human misery. The much-denigrated Bradford Hill (non) criteria (29) recognized the importance of population distributions of disease, as did more elaborate formulations of the same basic principles by Susser (106) and others. The mapping of social, ethnic, gender, and other inequalities are key to epidemiology, beyond its concern (as a public health science) with inequity. For those epidemiologists who denigrate our discipline for engaging with social deprivation (107), it is perhaps worth considering that health inequalities for particular causes can favor the less powerful social groups (108, 109), and this provides at least as much evidence regarding disease etiology as does the more usual (but apparently uninteresting) concentration of misery on the expropriated.

Population distributions of disease are of importance to etiological epidemiologists because they provide a cornerstone for the appropriate triangulation of evidence (72, 73, 84): deliberately gathering evidence from sources producing (near) orthogonal biases to strengthen causal inference. Indeed, from Snow (110, 111), Goldberger (112, 113), Sydenstricker (114) , and Frost (115) onwards, the formal history of epidemiology has involved drawing mental (or physical) maps of how the underlying external environment produced, through increasingly proximal processes, disease. In an innovative series of papers, Gerald Lower (116–118) developed an elaborate model of how molecular data could substantiate the causal nature of upstream socioenvironmental influences. The recent ability to generate such biological data at scale and to utilize germline genomics as a source of causal anchors (119) now allows these paths to be constructed. Thus, the effects of greater educational attainment on disease outcomes can be interrogated using quasi-experimental upstream perturbations (120, 121), and probabilistic causal chains leading to disease investigated. Multistep Mendelian randomization (122) can demonstrate how particular exposures influence the biology of the specific tissues of relevance to the disease being studied (123, 124), and the triangulation of evidence can include such highly compelling evidence of biological plausibility (another of Bradford Hill’s noncriteria (29)).

Recent advances in biological knowledge also throw light on the multitude of stochastic processes likely involved in human development and disease (45, 125). Indeed, it is the fact that, at an individual level, chance plays a considerable role in who gets disease, while at the aggregate population level, risk can be sharply defined, that underlies fundamental aspects of epidemiologic theory (45, 126). Geoffrey Rose’s influential notion that the determinants of the incidence rate experienced by a population may explain little of the variation in risk between individuals within the population (127)—that sick individuals and sick populations require different explanatory models—is indeed difficult to rationalize without this understanding (45, 48).

To conclude with a final example, readers can imagine what Morris would make of papers claiming substantial reductions in mortality consequent on religious service attendance (128). The apparent reductions in total mortality consequent on service attendance are larger than the differences by smoking reported by Doll and Hill in the seminal prospective British Doctors’ Study in 1956 (129). The exposures have the same relative distribution (the “protective” not smoking and service attendance having roughly the same prevalence in the populations under study). However, as Morris noted (1), all-cause mortality in the United Kingdom showed unfavorable trends in mortality among men as smoking levels increased in the population. In contrast, mortality rates in many countries showed unprecedented improvements during periods in which there were very substantial reductions in religious service attendance. The counterbalancing effects must, at a population level, be larger than any identified single cause (e.g., considerably larger than smoking). The fact that advanced causal inference methods were applied to the religious attendance study, including detailed sensitivity analysis, and yet no consideration was paid to underlying trends in the exposure or outcome under study (all-cause mortality) exemplifies modern epidemiology (130).

The central tenets of this commentary have been better articulated by many others (105, 115, 131–134) yet have had relatively little discernible influence on the discipline. As has been suggested, the views I express here may well reflect the last spasms emitted by a redundant and diminishing group refusing to recognize its superfluousness (135). I maintain that reflecting on how previously recalcitrant problems became solvable is a solid ground for advancing epidemiology, however. In addition to biology—from germline and somatic genomics through a cascade of mediating and interacting, including microbiological, processes—environmental monitoring, digital data collection, wearables, ingestibles (136), and more could be the drivers of future problem-solving. Connecting population health (with its understanding of broader social, economic, historical, geographic, and physical environmental influences) with methodological developments would allow epidemiologists to escape the fate Pearce envisaged (103), of us becoming phlebotomists for molecular biologists. As Bruno Latour explained in a different context, the argument that we should become postmodern is predicated on the false belief that we ever managed to assimilate modernity (137). Engagement with transformative understandings from other disciplines would allow etiological epidemiology to finally become modern.

## Abbreviations

CHD: coronary heart disease
HDL: high-density lipoprotein
NSE: nonshared environment
RCT: randomized controlled trial
